# Patient-reported disease knowledge and educational needs in Lynch syndrome: findings of an interactive multidisciplinary patient conference

**DOI:** 10.1186/1897-4287-12-1

**Published:** 2014-02-05

**Authors:** Sarah A Bannon, Maureen Mork, Eduardo Vilar, Susan K Peterson, Karen Lu, Patrick M Lynch, Miguel A Rodriguez-Bigas, YiQian Nancy You

**Affiliations:** 1 Department of Surgical Oncology, The University of Texas M. D. Anderson Cancer Center, Houston, Texas, USA; 2 Department of Gastroenterology, Hepatology, and Nutrition, The University of Texas M.D. Anderson Cancer Center, Houston, Texas, USA; 3 Department of Clinical Cancer Prevention, The University of Texas M.D. Anderson Cancer Center, Houston, Texas, USA; 4 Department of Behavioral Science, The University of Texas M. D. Anderson Cancer Center, Houston, Texas, USA; 5 Department of Gynecologic Oncology, The University of Texas M. D. Anderson Cancer Center, Houston, Texas, USA

**Keywords:** Hereditary nonpolyposis colorectal cancer (HNPCC), Genetic counseling, Patient education, Health education, Cancer surveillance, Lynch syndrome

## Abstract

**Background:**

Patients with Lynch Syndrome, the most common hereditary colorectal cancer syndrome, benefit from genetic education and family counseling regarding diagnostic testing and cancer surveillance/prevention recommendations. Although genetic counseling is currently the most common venue where such education and counseling takes place, little is known about the level of disease knowledge and education needs as directly reported by patients and families with Lynch Syndrome. Furthermore, experiences with forums for larger-scale knowledge transfer have been limited in the current literature.

**Methods:**

We conducted a one-day interactive multidisciplinary patient conference, designed to complement individual genetic counseling for updating disease knowledge, supportive networking and needs assessment among Lynch Syndrome patients and their family members. The patient conference was designed utilizing the conceptual framework of action research. Paired pre- and post-conference surveys were administered to 44 conference participants anonymously to assess patient-reported disease knowledge and education needs.

**Results:**

A multidisciplinary team of expert providers utilized a variety of educational formats during the one-day conference. Four main focus areas were: genetic testing, surveillance/prevention, living with Lynch Syndrome, and update on research. Thirty-two participants (73%) completed the pre-conference, and 28 (64%) participants completed the post-conference surveys. Nineteen respondents were affected and the remaining were unaffected. The scores of the disease-knowledge items significantly increased from 84% pre- to 92% post-conference (*p* = 0.012). Patients reported a high level of satisfaction and identified further knowledge needs in nutrition (71%), surveillance/prevention options (71%), support groups (36%), cancer risk assessment (32%), active role in medical care (32%), and research opportunities (5%).

**Conclusion:**

Our experience with a dedicated patient education conference focused on Lynch Syndrome demonstrated that such an educational format is effective for updating or reinforcing disease knowledge, for identifying patient-reported unmet educational needs, as well as for peer-support.

## Background

Lynch Syndrome (LS) is the most common inherited colorectal cancer (CRC) syndrome. Its hallmarks include germline defects in the DNA mismatch repair pathway and microsatellite-high tumor phenotype. Patients are most commonly predisposed to developing CRC, with reported lifetime risks ranging from 20-80%. [1,2] They are also at risk for multiple extra-colonic cancers, including endometrial, ovarian, gastric, small bowel, hepato-pancreatic-biliary, genitourinary, sebaceous skin, and glioblastoma [3-5]. Therefore, early diagnosis, multi-organ cancer surveillance, and risk-reducing preventive strategies represent the cornerstones of management in LS [6,7].

Patients and families benefit from education and counseling regarding their disease and regarding these key areas of care [8]. Indeed, lack of patient knowledge about their disease, cancer risks and treatment options, has been identified as a significant barrier to adoption of recommended care [9,10]. Currently, genetic counseling is the most common platform wherein the counselor transfers knowledge of the natural history, mode of transmission, and risks of a genetic disorder to patients and families. It also represents the main venue for discussing cancer risk management including recommended cancer surveillance and risk-reducing strategies, along with psychosocial support. However, little is known about the level of disease knowledge and education needs as directly reported by patients and families with Lynch Syndrome.

Furthermore, experiences with forums for larger-scale education regarding Lynch Syndrome have been limited in the current literature. Genetic counseling is an individual communicative process, and the availability and access to genetic counseling services can be limited [11,12]. Patients may have ongoing needs for disease education, beyond the initial genetic counseling session held most commonly at the time of initial diagnosis; they may benefit from reinforcing or updating disease knowledge and/or management recommendations. Furthermore, dissemination of genetic information and care recommendations to potentially at-risk family members can be difficult through the current one-time individual model of genetic counseling.

We conducted a disease-specific patient-oriented education conference to complement individualized genetic counseling services. The conference aimed to assess patient needs through interactive feedback, and to allow larger-scale knowledge transfer to patients and families, while also assembling patient-based supportive networks. We herein describe our findings in patient-reported disease knowledge and education needs, along with our experience with the organization, delivery, and educational impact of such a patient conference.

## Methods

### An action research framework for conference organization and development

In order to ensure the patient-centeredness of the LS patient conference, a conceptual framework of action research was adopted to maximize reciprocal interactions throughout the development, design, and delivery of the conference [13]. Action research is a broad style of research that has been widely applied in the field of education. In the teaching context, the goal is to gain knowledge from students that can lead to new ideas and strategies and that are then directly help to promote student success [14]. When adapted to the setting of our conference, action research represents a cyclical process of inquiry-and-feedback interactions among organizers and participants, leading to incorporation of participant input and informed actions, for the ultimate goal of better understanding and addressing the needs of patients and families with LS.

The Clinical Cancer Genetics (CCG) Program at the University of Texas MD Anderson Cancer Center (UTMDACC) established a Lynch Syndrome Patient Education Conference planning committee. The committee members included two genetic counselors, one behavioral scientist, and three physicians. The development of this conference occurred through two cycles of action research with participant input. During the first cycle, patient interests and needs as reflected from daily clinical interactions were discussed. Based on these, the target conference participants were defined, and included both affected and unaffected probands and family members. The overall aims of the conference were formulated as: (1) to directly collect patient-reported experiences, needs, and feedback related to clinical genetics care; (2) to provide patients and families with updated knowledge in the clinical care and research related to LS; (3) to connect patients and their families with the UTMDACC CCG Program and other experts in the field; and (4) to empower and connect patient/families with each other for sharing resources of support in their communities. During the second action research cycle, a convenience sample of five patients who presented for clinic visits was asked about their preferences and concerns regarding the logistics of the conference such as timing, location, duration and formats. Institutional grant funding allowed for free registration, and the conference location was set to be within car-travel distance of the institution, coinciding with the 4^th^ Biennal Meeting of the International Society for Gastrointestinal Hereditary Tumors (InSIGHT) held in San Antonio, Texas.

### Conference content and description

A single-day delivery format, spanning over a total of 6 hours, was chosen based on ease of travel. The educational format of the conferences included didactic sessions with either a single speaker or a panel of speakers, moderated round-table breakout sessions, as well as time for one-on-one question and answers. Didactic content and conference organization were summarized in Table 1. Topics covered by the six didactic sessions included: Historical perspective; Genetic testing; Surveillance; Psychosocial aspects of living with LS; and Recent/New research in LS. The lunch hour was utilized for a set of moderated round-table breakout sessions; each roundtable addressed a particular topic of interest to patients (Table 1).

**Table 1:** Summary of the didactic content and organization of the University of Texas MD Anderson Cancer Center Lynch Syndrome Patient Education Conference

**Time**	**Didactic content**	**Educational format**	**Speaker**
Morning	1. Historical perspective of Lynch syndrome	Lecture	Gastroenterologist
	2. Updates in genetic testing	Lecture	Genetic counselor
	3. Gastrointestinal surveillance strategies	Lecture	Colorectal surgeon
	4. Gynecologic surveillance strategies,	Lecture	Gynecologic surgeon
	5. Living with Lynch syndrome	Lecture & Volunteer patient panel	
	a) Psychosocial aspects		Behavioral scientist
	b) The Genetic Information Non-Discrimination Act (GINA)		Genetic counselors
	c) Patient perspectives of the disease		Volunteer patients
Lunch	Round-table topics included: “I am new to Lynch”; “I am a survivor”; Healthy lifestyle”; “Genetic testing and GINA”	Round-table breakout sessions with 1–2 speakers and 8–15 attendees	All speakers
Afternoon	6. Advances in Lynch syndrome: update on research. (Emerging chemoprevention trials)	Lecture	Colorectal surgeon & Invited expert
	Individualized question & answer	Free format	All speakers

The conference speakers included volunteer patients, as well as colorectal surgeons (M.A.R-B and Y.N.Y.), behavioral scientists (S.P.), gastroenterologists (P.M.L.), gynecologic oncologists (K.L.), genetic counselors (S.A.B.) and invited experts (Professor Sir John Burn, Professor of Clinical Genetics, Newcastle University, United Kingdom). All written materials including speaker slides were organized, bound and provided to participants free of charge.

### Participants

The institutional genetic counseling database was queried approximately 6 months prior to the anticipated conference date for potential participants. We included probands and family members with a defect (either pathogenic mutation or likely deleterious variant) in any DNA mismatch repair gene. We also included probands and family members with non-sporadic microsatellite-high phenotype tumors (i.e. absence of *MLH1* promoter methylation or *BRAF*-V600E mutation) in whom either no pathogenic mutation was found on germline testing or recommended genetic testing had been declined. These target individuals were mailed save-the-date invitations and a flyer with preliminary conference agenda 1 and 3 months prior to the conference. Invitations were extended to all family members and were disseminated through the institutional public website, and other LS-focused organizations and their corresponding websites such as The Daily Strength Lynch syndrome group (http://www.dailystrength.org/groups/lynch-syndrome), The Colon Club (http://www.colonclub.com), Lynch Syndrome International (http://lynchcancers.com), and CCARE for Lynch Syndrome (http://www.fightlynch.org). Participants self-registered for the Education Conference via the CCG Program public website. There was no cost for conference registration.

### Conference survey

At the time of the conference, all participants were given a set of paired pre- and post-conference surveys along with their conference materials. Participants self-selected to complete these anonymous surveys. A consent form was attached to the front of the surveys, and participants acknowledged their voluntary consent by electing to complete the surveys. The pre-conference survey was administered and collected prior to the start of the conference program. Participants were asked not to uncover the post-conference survey until after the entire conference program had concluded. The post-conference survey was completed and collected as participants departed. The UTMDACC Institutional Review Board granted exempt status to this study.

The paired pre- and post-conference surveys were designed *a priori*. They were pilot tested by two physicians and a graduate research student who were not involved in conference planning. The pre-conference survey comprised 35 items. The post-conference survey comprised 31 items (detailed in Table 2). Twenty minutes were allotted to each survey. The pre-conference survey assessed demographic and health characteristics; personal experience with LS including cancer history, surgical and other treatments, surveillance practices and disease knowledge sources; and 12 knowledge assessment items covering different aspects of LS relevant to the conference didactic sessions. All items on the pre-conference survey were of the multiple-choice format; the 12 items were scored from 0-100%, with higher percent score indicating a greater proportion of correct answers. The post-conference survey contained the 12 knowledge assessment items again, but their order was reorganized to minimize test/re-test bias. We also assessed patient-reported knowledge needs; participants were asked to indicate which health topics they felt needed more knowledge. Participants were also asked open-ended questions regarding educational needs that were most adequately addressed by the conference as well as any other unaddressed educational needs.

**Table 2:** The University of Texas MD Anderson Cancer Center Lynch Syndrome Patient Education Conference patient survey

**Question domains**	**Pre-conference survey**	**Post-conference survey**
1. Respondent demographics	7 items (Multiple-choice)	0 item
	● Age at LS diagnosis	
	● Demographics: age, gender, race/ethnicity	
	● Marital status, educational background, and health insurance status	
	● Method of diagnosis	
2. Patient-reported experiences with LS	13 items (Multiple-choice)	0 item
	● Personal history of cancers diagnoses	
	● Personal history of surgical interventions	
	● Surveillance for LS-related cancers	
	● Sources of knowledge regarding LS	
3. Disease knowledge	12 items (Multiple-choice)	12 items (Multiple-choice)
	● Hereditary basis of LS	● Hereditary basis of LS
	● Transmission pattern	● Transmission pattern
	● Risks of LS-related cancers	● Risks of LS-related cancers
	● Surveillance strategies	● Surveillance strategies
	● Prophylactic options	● Prophylactic options
4. Patient needs assessment and feedback	0 item	19 items (Rating, multiple-choice, free text)
		● Learning needs addressed by the conference
		● Patient-reported further learning needs
		● Feedback regarding conference

### Data analysis

Descriptive statistics were used to summarize survey items addressing demographics, experience with LS, and perceived value of the conference. All continuous variables were described by mean and standard deviation (SD), while all categorical variables, by number and percentage. Gender-specific items were summarized according to self-reported gender information. Twelve survey items examining disease-specific knowledge were compared between pre- and post-conference surveys using paired t-test. All statistical tests were two-sided and statistical significance was denoted by p-value <0.05. All analyses were performed using STATA statistical software (StataCorp. 2007. *Stata: Release 10.* College Station, TX).

## Results

Forty-four individuals attended the conference. Thirty-two (73%) completed the pre-conference survey and 28 (64%) participants completed the post-conference survey.

### Demographics (Pre-conference survey, n = 32)

The majority of the respondents was female, white, educated, married, and had health insurance (Table 3). Nineteen (59%) respondents reported a personal diagnosis of LS. The remaining respondents were family members without LS (Table 3).

**Table 3:** Demographic characteristics of the 32 participants who returned the pre-conference survey

**Characteristic**	**Number of patients (% )**
Age, years, mean	54
Female	20 (63)
Race/Ethnicity	
White	27 (84)
Hispanic	5 (16)
Black/Asian/Other	0 (0)
Education level	
College/Post-Graduate	22 (69)
Vocational/Some college	1 (3)
High school	9 (28)
Marital status	
Married	26 (82)
Never married	3 (9)
Divorced/Separated/Widowed	3 (9)
Insurance status	
Insured	32 (100)
Not insured	0 (0)
LS Status	
Personal history of LS	19 (59)
Family history of LS	13 (41)
FDR with LS	3
Other relative with LS	1
Non-blood relative with LS	4

FDR = first degree relative.

LS = Lynch syndrome.

### Patient-reported experience with LS (Pre-conference survey, n = 19 with LS)

Among the 19 respondents with LS, 12 (63%) were female and the mean age at LS diagnosis was 47 years (SD: 8 years; range: 22–71). Diagnosis was based on presence of pathogenic mutation in 14 (74%), non-sporadic microsatellite-high tumor phenotype in 4 (21%), and pedigree criteria in 1 (5%).

Respondents most commonly reported a personal history of colorectal polyps (68%) or cancer (53%), followed by small bowel polyps (5%) or cancer (5%), and sebaceous skin neoplasm (5%). Among 12 women with LS, 5 (42%) had endometrial cancer and one (8%) ovarian cancer. Eight (42%) of the 19 LS patients had undergone colorectal surgery, and 11 (92%) of the 12 female LS patients had gynecologic surgery.

Regarding multi-organ cancer surveillance, 18 (95%) reported undergoing colonoscopy, and the frequency was every 1–2 years in 11 patients, every 3 years in one, and unspecified in the remaining. Upper gastrointestinal endoscopic surveillance was reported by 13 (68%) at a frequency of at least every 1–2 years in 8 patients, and unspecified in the remaining. Surveillance of urinary tract cancers by urinalysis was reported by 10 (53%), and the frequency was annual in 6 patients. Among women with LS, 9 (90%) reported endometrial surveillance at least every two years while their uterus were in place.

Respondents indicated that their main source of knowledge regarding LS came from the internet (57%), their health care providers (45%), written materials (30%) and medical journals (25%). Among healthcare providers, they indicated genetic counselors (54%) as the main source of information, followed by gastroenterologists (23%) and/or surgeons (11%).

### Disease knowledge (Paired pre- and post-conference survey, n = 28)

Twenty-eight paired pre- and post-conference surveys were compared for scores from the 12 disease knowledge questions (Table 4). The mean knowledge scores improved from 84.2 (SD: 9.6) pre-conference to 91.7 (SD:6.7) post-conference (*p* = 0.012). For each of the questions, the proportion of patients who answered the particular question correctly increased from pre- to post-conference: the median increase among all questions was 7.4% (SD: 7.4%; p = 0.025; Table 4). The single item with the greatest improvement was regarding the use of tumor molecular testing to screen for patients with Lynch syndrome (question 10).

**Table 4:** Proportions of patients answering correctly to each of the twelve disease knowledge questions from pre- to post-conference surveys

	**Disease knowledge question**	**Patients correctly answering**		**% Improvement in proportions of patients answering correctly**
		**Pre-conference: no. (%)**	**Post-conference: no. (%)**	
1	Lynch syndrome can be passed to a child through the mother or the father	28 (100)	28 (100)	0.0
2	Within a family, Lynch syndrome can affect each family member differently	26 (93)	28 (100)	7.1
3	Colorectal cancer is the only type of cancer that happens more often in people with Lynch syndrome	24 (86)	23 (82)	−3.6
4	If you look like your parent who has Lynch syndrome, you are more likely to have Lynch syndrome yourself	21 (75)	24 (86)	10.7
5	If a parent has Lynch syndrome, each child will have a 1 in 4 (or 25%) chance of having Lynch syndrome	20 (71)	23 (82)	10.7
6	On average, people with Lynch syndrome have a 60-80% chance of developing cancer of the colon or rectum	23 (82)	26 (93)	10.7
7	People with Lynch syndrome should have a scope exam of their colon or rectum every 1–2 years	26 (93)	28 (100)	7.1
8	If a person with Lynch syndrome has his/her entire colon surgically removed, he/she no longer needs continued surveillance or evaluation of the remaining rectum or pouch	25 (89)	27 (96)	7.1
9	Women with Lynch syndrome have up to a 60% chance to develop uterine/endometrial cancer	25 (89)	26 (93)	3.6
10	Tests performed on the colon or uterine tumor tissues can be used to help diagnose Lynch syndrome	19 (68)	26 (93)	25.0
11	Currently, there is only one gene known to be associated with Lynch syndrome	22 (79)	25 (89)	10.7
12	There is a blood test available that can often identify the genetic cause of Lynch syndrome	24 (86)	24 (86)	0.0

### Participant-reported learning needs and feedback (Post-conference survey, n = 28)

Respondents indicated that the conference was the most helpful in fulfilling their learning needs through: “speaking with providers/experts”, “networking with other families with LS”, and “receiving a book of the presentation slides for future reference”.

**Figure 1:**
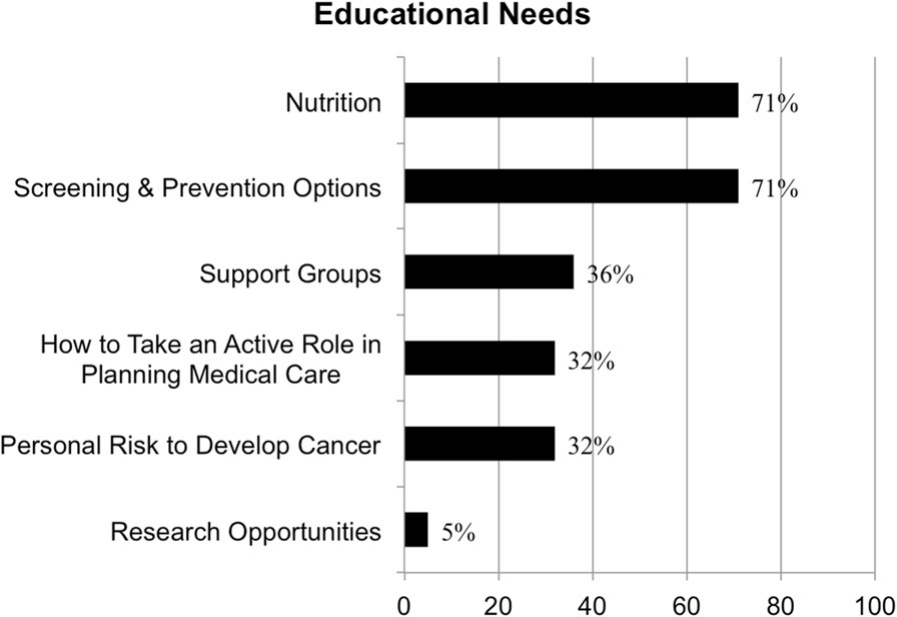
Education needs as reported by Lynch syndrome patients.

The most commonly indicated areas of additional learning needs and questions included: nutrition and impact on disease (71%), surveillance and prevention options (71%), support groups (36%), risk for developing certain types of cancers/my family’s risk for developing cancer (32%), active role in planning my medical care (32%) and research opportunities (5%; Figure 1).

A high level of satisfaction was reported by respondents who indicated strong agreement with the following statements: “I learned a lot of useful information about Lynch syndrome today” (28 respondents, 100%); “I plan to use information I learned today when making decisions about my healthcare” (27, 96%); “I would recommend this conference to others” (27, 96%); “I feel the conference was the right length” (27, 96%); and “I would attend this conference again if updated information were provided” (26, 93%).

## Discussion

Patients with hereditary cancer syndromes such as LS benefit from cancer risk assessment, disease knowledge, and cancer surveillance and prevention. Currently, these tasks are most commonly accomplished through one or more individualized genetic counseling sessions. Patient-reported disease knowledge and educational needs have not been directly reported in the literature. Furthermore, potential unmet needs in LS may include addressing knowledge needs of family members, continued updating of disease knowledge over time, assessing patient needs and feedback in aggregate form, as well as opportunity for group support and networking. Yet experience with educational formats beyond individual genetic counseling has not been widely published. We adopted the conceptual framework of action research and delivered a patient education conference for LS, borrowing experiences from similar conferences for patients with hereditary breast and ovarian cancer syndromes [13]. We found that such a conference highlighted current disease knowledge and unmet educational needs, allowed effective large-scale knowledge transfer, and was associated with a high level of patient satisfaction. These reported findings will hopefully encourage organization of similar conferences in the future and stimulate further research regarding optimal ways for health education in patients with LS.

One goal of our disease-focused patient education conference was to directly collect patient-reported experiences, needs, and feedback, while also providing patients and families with updated knowledge in the clinical care and research related to LS. While patient conferences with similar goals have been reported in the HBOC population [13], to our knowledge, there has been no formal report of such conference for LS. We found that patients and family members attending our conference possessed a relatively high level of disease knowledge at the onset. Despite this, a focused day-long conference offering multi-faceted education through multiple formats still led to a significant improvement in disease-specific knowledge beyond the baseline level. Importantly, our patients identified healthcare providers, and in particular, genetic counselors, as an important source of their knowledge about their disease. Thus, we suggest that patient conference appears to provide additional educational benefit from patient conference that is built on the foundation of prior individual genetic counseling. The conference serves to update and reinforce, but not replace, genetic counseling as an effective avenue for patients to learn about their hereditary syndrome.

Through direct solicitation, we have also documented learner-reported educational needs and topics of particular interest to patients and families with LS. Areas of learning needs included: nutrition and its impact on disease, options for cancer surveillance and prevention, support groups, and how to take a more active role in medical care. Eliciting participant feedback in these areas of need greatly informs future research, as these are the target areas for information in both future conferences and in future individual counseling sessions. These can also form contents of patient education pamphlets, educational websites, and/or other education venues, particularly in the view of the fact that patients identified the Internet as the most common source of information related to their disease.

In addition to education, the patient conference was designed to allow for maximal and efficient interactions among a multidisciplinary team of providers and patients/families, among patients and members of their own families, and among all individuals and families affected by the same disease. Interactive opportunities included volunteer patient panels, breakout round tables, question-and-answer sessions, as well as free times. By having providers interact with groups of patients, rather than one-on-one, the discussions triggered by individual concerns could become educational to and shared with the whole group, thereby improving educational efficiency. Additionally, by gathering healthcare providers with diverse expertise together, patient questions and concerns could be addressed by the most suitable and experienced provider while still allowing input from other providers and patients. Most importantly, for patients with rare syndromes such as LS, enabling peer-support and networking among affected families fosters the building of a supportive community. Based on patient-reported experiences with LS recorded herein, variations existed among the types of cancers experienced by the respondents, whether patients had undergone surgical procedures, and among the various surveillance practices. This rich mixture of experiences provides the raw material for patient-patient interactions and exchanges. A sense of collective community often helps to provide psychological support to patients with rare hereditary syndromes such as LS, as reflected by the growth and success of institutional/national registries and support groups such as Lynch Syndrome International. Indeed, more than a third of the respondents identified support groups as an unmet need in their care. The conference format can serve as a springboard for formation of such groups. With increasing use of social media and internet-based communications, virtual forms of such conferences might be developed in the future.

We have reported an early and single-institutional experience with patient education in LS, and our report is limited in several ways. Our participants represent a clinic-based population from a single geographic region with unique demographic characteristics (Table 3), thus limiting the generalizability of our experience and findings. Secondly, respondent’s knowledge regarding LS was assessed by short-term recall only. Long-term follow-up data would be needed to assess knowledge retention or knowledge-based changes in health-related behavior. Thirdly, because the surveys were anonymous, we could not assess for responder vs. non-responder bias.

In conclusion, an interactive multidisciplinary patient conference provided an opportunity for direct assessment of patient-reported knowledge and education needs in LS. This reported experience contributes to the larger effort to provide comprehensive care to patients with hereditary cancer syndromes. Further research is needed to examine the long-term impact of such an educational model in terms of knowledge retention, guideline adoption, and translation into improved patient outcomes.

## Abbreviations

HNPCC: Hereditary nonpolyposis colorectal cancer; LS: Lynch syndrome; CRC: Colorectal cancer; CCG: Clinical Cancer Genetics; UTMDACC: University of Texas MD Anderson Cancer Center; SD: Standard deviation.

## Competing interests

The authors declare that they have no competing interests.

## Authors’ contributions

**a.** Substantial contributions to conception and design (SKP, KL, PML, MAR, YNY), or acquisition of data (SAB, MB), or analysis and interpretation of data (SAB, EV, YNY); **b.** Drafting the article (SAB, YNY) or revising it critically for important intellectual content (SKP, KL, PML, MAR, EVS, MB); **c.** Final approval of the version to be published (All authors).
